# A principle‐based framework for disclosing a psychosis risk diagnosis

**DOI:** 10.1111/bioe.13106

**Published:** 2022-11-09

**Authors:** Oliver Y. Zhang, Doug McConnell, Adrian Carter, Jonathan Pugh

**Affiliations:** ^1^ Faculty of Medicine, Nursing and Health Sciences Monash University Melbourne Victoria Australia; ^2^ Oxford Uehiro Centre for Practical Ethics, Faculty of Philosophy University of Oxford Oxford UK; ^3^ Turner Institute for Brain and Mental Health Monash University Melbourne Victoria Australia

**Keywords:** at‐risk mental state, autonomy, diagnosis, disclosure, psychosis risk, ultra‐high risk

## Abstract

In recent decades, researchers have attempted to prospectively identify individuals at high risk of developing psychosis in the hope of delaying or preventing psychosis onset. These psychosis risk individuals are identified as being in an ‘At‐Risk Mental State’ (ARMS) through a standardised psychometric interview. However, disclosure of ARMS status has attracted criticism due to concerns about the risk–benefit ratio of disclosure to patients. Only approximately one quarter of ARMS patients develop psychosis after three years, raising concerns about the unnecessary harm associated with such ‘false‐positive’ results. These harms are especially pertinent when identifying psychosis risk individuals due to potential stigma and discrimination in a young clinical population. A dearth of high‐quality evidence supporting interventions for ARMS patients raises further doubts about the benefit accompanying an ARMS disclosure. Despite ongoing discussion in the bioethical literature, these concerns over the ethical justification of disclosure to ARMS patients are not directly addressed in clinical guidelines. In this paper, we aim to provide a unified disclosure strategy grounded in principle‐based analysis for ARMS clinicians. After considering the ethical values at stake in ARMS disclosure, and their normative significance, we argue that full disclosure of the ARMS label is favoured in the vast majority of clinical situations due to the strong normative significance of enhancing patients' understanding. We then compare our framework with other approaches to ARMS disclosure and outline its limitations.

## 1 INTRODUCTION

In recent decades, researchers have attempted to prospectively identify individuals in the prepsychotic phase of schizophrenia in order to prevent or delay psychosis onset.^1^ Individuals at risk of psychosis present with a mixture of both attenuated psychotic symptoms (such as delusions, hallucinations^2^ or disorganised speech) and other nonpsychotic features, including mood symptoms like depression or anxiety, subjective clinical distress and functional impairment.^3^ Individuals meeting a certain threshold after assessment by specialist clinicians through a psychometric interview known as the Comprehensive Assessment of At‐Risk Mental States (CAARMS)^4^ can be labelled with the term ‘At‐Risk Mental State’ (ARMS),^5^ signifying their significant risk for developing psychosis.^6^

However, there are several ethical challenges associated with the disclosure of psychosis risk.^7^ Central to these concerns are questions about the risk–benefit ratio of disclosure to these patients. Heterogeneity in clinical outcomes and a low transition rate to psychosis have resulted in some questioning whether the psychosis risk paradigm can be meaningfully applied in clinical practice at all.^8^ These claims are grounded in concerns over a high false‐positive rate, with only 26% of ARMS patients developing psychosis over 3 years.^9^ Patients often develop another comorbid mental illness, maintain their subthreshold psychotic symptoms or recover completely.^10^

Theorists have also raised concerns over the potential harms of labelling psychosis risk patients, such as stigma and the potential for patients to be unnecessarily exposed to harmful antipsychotic medication.^11^ Critics of the psychosis risk paradigm further point to a lack of high‐quality evidence on interventions for ARMS patients.^12^ If an ARMS diagnosis does not result in effective intervention, then disclosure may only provide the patient with a potentially vague, obfuscating and distressing label.^13^

Despite these concerns, there is minimal ethical direction in ARMS clinical guidelines assisting clinicians with disclosure. Australian Early Psychosis guidelines recommend that ‘information about the level of risk should be carefully provided taking into account social, educational and cultural factors’ without further direction regarding how to navigate complex cases.^14^ Canadian guidelines provide well‐reasoned clinical recommendations for management, but do not engage with the ethical considerations in ARMS disclosure, and do not provide an ethical rationale for their adopted recommendations.^15^ European guidelines recommend that ARMS criteria ‘should only be used and communicated with utmost care in children and young adolescents’ without specifying how or why these patients should be disclosed information in a different manner.^16^ Meanwhile, a 2019 study found that French psychiatrists were reluctant to disclose their patients' psychosis risk or refer them for standardised assessments like the CAARMS,^17^ suggesting that the ethical disclosure of psychosis risk is an area that would benefit from further normative analysis. This paper attempts to clarify the ethical justification for disclosure in psychosis risk patients through principle‐based analysis. We build on previous ethical analysis that has identified key ethical values at stake in disclosure^18^ by highlighting some of the complexities in applying and weighing these principles. Using this analysis, we outline a flow‐chart decision aid that may assist clinicians in developing an ethically grounded disclosure strategy in particular cases.

## 2 ETHICAL PRINCIPLES AND PSYCHOSIS RISK DISCLOSURE

### 2.1 Respect for autonomy by enhancing understanding

The principle of respect for autonomy provides strong justification for disclosing material medical information to patients. Indeed, in the context under consideration here, Corcoran has suggested that filtering the information provided to patients is not compatible with the principle of patient autonomy.^19^ It is important to distinguish two reasons why this may be so. One reason for this is that disclosure is typically necessary for individuals to achieve the *understanding that autonomous decision‐making requires*. Another secondary autonomy‐based justification is that the individuals may have an autonomous preference to know this information. We shall consider each justification in turn; however, we shall also suggest that considerations of autonomy do not always unambiguously favour information disclosure.

#### 2.1.1 The obligation to disclose to enhance understanding

Sufficient understanding is necessary for autonomous decision‐making.^20^ In a clinical context, due to the asymmetry in medical knowledge between a clinician and patient, acquiring this understanding is usually achieved through clinician disclosure. Indeed, this is reflected in legal standards of information disclosure; clinicians have an obligation to disclose information that is deemed to be material to the patient's treatment decision. One justification for such requirements is that patients need to adequately understand their options if they are to make decisions in accordance with their values. Patients may also need to understand diagnostic information to make autonomous nonmedical decisions about their life beyond the clinical encounter.

This naturally raises questions about the quantity and quality of information that should be disclosed; what information is material? Although legal standards of disclosure may be predominantly in place to demarcate the scope of the clinician's duty of care, rather than to determine the degree of understanding that autonomous decision‐making requires, they can nonetheless provide insight into information that should be disclosed to patients to facilitate their autonomy.^21^ In their discussion of disclosure of APS, Mittal et al. advert to the ‘reasonable person’ and ‘reasonable doctor' standards that are evident in U.S. law.^22^ Notably, they go on to suggest that it is not clear that there is an obvious legal obligation to disclose information about a *research diagnosis* that is not widely accepted by the medical community.^23^ Accordingly, they suggest that unless treatment for the condition in question is required, there may be some scope for clinicians to exercise a degree of discretion about whether to disclose a research diagnosis.^24^ Following the Montgomery ruling in the United Kingdom, which notably uses a ‘particular patient’ prong in its hybrid materiality test (according to which information should be disclosed if the physician should reasonably be aware that the particular patient wants to know the information in question),^25^ there is ongoing debate about which standard of disclosure should determine legal obligations pertaining to diagnoses.^26^

However, while considerations of autonomy provide a strong prima facie ethical justification to disclose, there is a need for caution here. Factors such as clinical heterogeneity and a high false‐positive rate raise important challenges to the claim that full disclosure of ARMS status will straightforwardly enhance a patient's understanding.

#### 2.1.2 Would psychosis risk disclosure enhance understanding?

One concern in this context that Corcoran highlights in her analysis is that ‘… it may be difficult for patients and their families to appreciate the difference between a susceptibility and a disease’, and there is a concern that a suggestion of vulnerability might be mistaken for a quasi‐diagnosis.^27^ This concern is particularly worrisome, given that most ARMS patients do not develop psychosis, with only 26% transitioning to psychosis after 3 years.^28^ A high proportion of ARMS patients also fulfil criteria for comorbid nonpsychotic disorders, such as depression and anxiety, both at the time of ARMS diagnosis (70.3%) and at the 6‐year follow‐up (56.3%).^29^ Framed in this way, one may conclude that an ARMS label may seem more predictive of patients developing mental disorders other than psychosis.

However, these statistics fail to underscore how fulfilling ARMS criteria signifies a patient's risk *specifically for psychosis*. This is most evident when considering that ARMS‐positive patients have a 60‐fold higher risk of developing psychosis (26% within 38 months) than the general population (0.43%), while an ARMS‐negative^30^ outcome results in a significantly lower 1.56% risk of psychosis.^31^ Additionally, a long‐term validity study found that being ARMS positive was associated with a *lower* risk of developing nonpsychotic disorders compared with individuals who tested ARMS negative.^32^

In addition, 95% of patients actively *experience* attenuated psychotic symptoms^33^ in addition to experiencing functional decline and clinical distress similar to other coded psychiatric disorders.^34^ Disclosure of ARMS status can thereby enhance patients' understanding of these symptoms so that they can make informed decisions about treatment and other nonmedical life decisions.

There is also a possibility that developing psychosis may compromise or negatively influence an ARMS patient's future decision‐making capacity. One key distinction between frank psychosis and ARMS patients is that psychotic patients lack insight, while ARMS patients retain insight.^35^ Insight here refers to an individual's ability to recognise his or her own mental state, thoughts and actions as abnormal.^36^ Unsurprisingly, systematic reviews have found that psychotic patients with poor insight are unlikely to possess decision‐making capacity.^37^ Therefore, disclosing a patient's ARMS label while they have decision‐making capacity^38^ may allow patients to make their own medical decisions in a timely fashion.

However, there is a need for caution here. Ensuring adequate understanding of risk information is a significant challenge in medicine, even when the condition in question is well understood.^39^ This challenge is exacerbated in the context of ARMS, since the medical understanding of ARMS and its implications for risk is still evolving.

#### 2.1.3 Factors that may impede disclosure from enhancing understanding

An important limitation to the autonomy‐based justification of the disclosure of medical information arises when a person lacks decision‐making capacity. It is important here to distinguish (i) capacity in the institutional sense, which denotes that an individual is legally recognised as having the authority to make a particular decision, and (ii) capacity in the noninstitutional sense, which denotes that the individual has the ability to make that decision autonomously.^40^ For example, those under the age of 16 may not be recognised as having legal capacity to refuse a beneficial medical treatment in the institutional sense (simply on the basis of their age), even though they may possess the abilities that are necessary for making that decision autonomously.^41^ Naturally though, when a patient lacks the capacity in the noninstitutional sense because, for instance, they are unable to understand information that is material to their decision, the autonomy‐based reasons for the clinician to disclose that information weaken and other principles may be taken to have greater moral significance.

Of course, all of this is compatible with the claim that there are still autonomy‐based reasons to enhance the understanding of patients who lack either form of capacity. In cases where a patient lacks both forms of capacity to make a particular decision, enhancing their understanding in keeping with their cognitive abilities enables clinicians to best respect the (perhaps limited) autonomy that is present in these patients. Facilitating their understanding as much as possible can enable them to assent to treatment through a shared decision‐making process, even if they cannot provide valid consent.^42^

A quite different obstacle is that there may also be contextual barriers to the sort of communication of a diagnosis that is conducive to facilitating patient understanding.^43^ Indeed, clinicians who are not ARMS specialists may not be able to provide detailed or evidence‐based information about psychosis risk even to patients who do have the capacity to understand that information. In itself, recognising and diagnosing a patient as ARMS is difficult because of the wide variance in patient presentation and a lack of defining clinical features.^44^ This is difficult even for youth mental health clinicians without using a standardised psychometric test like the CAARMS. One study, which assessed whether these clinicians (who were not formally trained in psychosis risk) could accurately identify ARMS status in an unstructured assessment, found that compared to the gold standard assessment (CAARMS), only 53% of ‘ARMS’ patients actually fulfilled ARMS criteria. These data speak in favour of providing further education and support about ARMS to clinicians working with these patients.^45^ If clinicians are to disclose a patient's risk state, they should have the appropriate clinical training to diagnose and disclose such information to each patient.^46^

Another way in which disclosure may not enhance understanding is if information is too complex for the patient to comprehend.^47^ Conveying risk information in a manner that maximises understanding is no small task when the goal is for patients to understand and have the opportunity to utilise the information associated with an ARMS disclosure. Indeed, the complexity of the relationship between disclosure and understanding has been recognised by authors investigating disclosure and participant understanding in clinical oncology research trials, where 64% of participants did not understand that trial participation carried additional risks to standard cancer treatment and 29% believed that the trialled treatments were ‘proven to be the best treatment for my type of cancer’.^48^

In light of these findings, one method to promote both disclosure and understanding is to convey ‘key information’ as a part of information disclosure, which places ‘emphasis on participant comprehension’.^49^ One way of applying this in ARMS disclosure may be through a simple description of ARMS prognosis, such as disclosing that broadly, ARMS individuals (over a 12‐month period) generally follow three courses: recovery of their symptoms (43.2%), ongoing attenuated psychotic symptoms (41%) or transition to psychosis (15.8%).^50^ This is a simplified, but functionally accurate description of the 17 different clinical trajectories for ARMS patients identified by Polari et al.

### 2.2 Respecting an individual's autonomous preference

A second autonomy‐based justification that can strengthen the normative reasons in favour of disclosure is that disclosure may respect an autonomous person's preference to know information about themselves. Notice that the question of whether a person *wants* to know some information is a separate issue to the question of whether knowing that information is necessary for them to make an autonomous treatment decision.

Superficially, one may assume that help‐seeking individuals who are referred to specialist clinics would have a preference to know their psychosis risk. Polari et al.'s study, which surveyed ARMS patients and caregivers regarding their preference for the timing and extent of psychosis risk disclosure, broadly supports this notion.^51^ All patients and caregivers in this study stated that they would prefer some information about their psychosis risk at some point in time, with 57% of patients stating that they would like the information ‘as early as possible’.^52^

An individual's right to be informed of medical information is enshrined in the ‘European Convention for the protection of Human Rights and Dignity of the Human Being with regard to the Application of Biology and Medicine’, Article 10(2).^53^ Crucially, the very same article of this significant legal instrument also enshrines a patient's right *not* to know certain information. The so‐called Right Not to Know (RNTK) has been widely discussed, particularly in the context of genetic information,^54^ and it is increasingly being invoked in legal appeals.^55^ Nonetheless, it has a complex relationship with autonomy. While there is an autonomy‐based argument in favour of respecting autonomous preferences to remain ignorant of certain information, it might be argued that the RNTK runs contrary to autonomy‐based arguments from the previous section, according to which disclosure is often required to ensure that patients have the degree of understanding necessary for autonomous decision‐making. Of course, this autonomy‐based objection to the RNTK has weaker force when there are concerns about the extent to which the information in question will serve to enhance understanding, as discussed above. Nonetheless, Andorno has suggested that an autonomy‐based RNTK may be justified once an individual meets a *threshold of understanding* to adequately make an autonomous decision.^56^

Empirical data support the claim that some psychosis risk patients may wish to invoke the RNTK. Polari et al. also found that some patients preferred ‘partial disclosure’ (15%) over full disclosure.^57^ In this context, partial disclosure involved disclosing an ‘ARMS’ label initially, but only providing an explanation for that label at a later time ‐ or in some cases, no explanation at all. Additionally, other researchers have found that affective symptoms (e.g., depression or anxiety) were *commoner* causes (47.1%) of help‐seeking behaviour in ARMS patients than sub‐threshold psychotic symptoms (39.8%).^58^ Patients who are more concerned about their affective symptoms may have a plausible interest in not knowing their *specific* psychosis risk if their primary complaint is well managed.

We believe that an RNTK in psychosis risk may be ethically justified^59^ under certain conditions. First, patients should be aware that they are at *some* risk of psychosis so that they have sufficient understanding to autonomously exercise their RNTK. Second, patients should be aware that clinicians can provide more detailed information about their psychosis risk if desired.

Of course, the right not to know raises particularly complex ethical and legal questions when the right is invoked with respect to information about heritable disorders, given the implications that disclosure can have for third parties. Indeed, there have been diverging legal judgements on this question in recent years. In the U.S. context, the judgement in Pate vs Threlkel suggests that a duty of care can arise to genetic relatives of a patient to warn about the heritability of a condition.^60^ However, in England and Wales, in ABC vs St George's trust, the court found that a clinician's duty of care did not extend to disclosing confidential diagnostic information about a genetically related patient.^61^ In view of these significant complexities for disclosure practices, we shall set these questions aside in our discussion. We also do so on the basis of our agreement with Mittal et al., who highlight the important difficulties facing estimates of the heritability of schizophrenia specifically.^62^

Having considered two autonomy‐based arguments in favour of disclosure, we now turn to arguments grounded by considerations of beneficence.

### 2.3 Beneficence

Since patients will only be able to access treatment if they know that they have a condition requiring it, diagnosis disclosure can promote beneficence when there is an efficacious treatment for their disorder. In the context of psychosis risk, while there appears to have been improvement in the standard of treatment,^63^ evidence at the level of systematic review for ARMS interventions is currently lacking.^64^

However, there are other reasons why disclosure may benefit an ARMS patient. A recent study found that ARMS patients had reduced psychological distress and a better sense of control over their health after their diagnosis was disclosed to them,^65^ a finding consistent with the reaction of other mental health patients where disclosure provided a source of explanation for their symptoms.^66^

Additionally, disclosure may result in better engagement with mental health services before patients may develop psychosis, which benefits ARMS patients whether they eventually develop psychosis or not. This is especially pertinent when considering the volatile and clinically uncertain prognosis of ARMS patients. Timely disclosure of an ARMS diagnosis not only acts as a unified source of explanation but also empowers patients to identify warning signs of deteriorating mental health, whether that be psychosis or another mental health disorder. Providing a diagnostic label to patients may also increase the resonance of clinical recommendations such as the cessation of cannabis use, as 27% of ARMS patients also fulfil criteria for a comorbid cannabis use disorder.^67^

Having outlined the arguments in favour of disclosure (and some of their attendant limitations), we now turn to nonmaleficence‐based arguments against disclosure.

### 2.4 Nonmaleficence: The therapeutic privilege

The principle of nonmaleficence is sometimes used to justify the so‐called ‘therapeutic privilege’.^68^ Bortolotti and Widdows capture the thrust of the therapeutic privilege in the context of schizophrenia as follows:

The idea is that knowledge of diagnosis and prognosis can make things worse for the client when the disorder is commonly stigmatised as untreatable or irreversible, and doctors should privilege the success of therapeutic interventions over other considerations.^69^

Corcoran also notes that the stakes of this issue may change in the context that we are considering here when patients are younger or less symptomatic. In such populations:

… the idea of being vulnerable for psychosis could leave the false positives with a lasting sense of being fragile, damaged or a little bit sick. It might alter their goals, or make them less likely to be achieved; it could be harder to find motivation for a future that threatens to be taken away by illness.^70^

Nonetheless, the therapeutic privilege in clinical practice should be carefully constrained for three reasons. First, while the clinician is qualified to judge a patient's best *medical* interests, they may not be privy to details of the patient's life and values.^71^ Therefore, invoking the therapeutic privilege is inherently paternalistic, and will often serve to significantly undermine autonomy by undermining the patient's understanding or by failing to respect their preference to be informed of medical information that concerns them. The therapeutic privilege should only be invoked if the harm prevented is sufficient to outweigh these costs to autonomy.

Second, even in more paternalistic eras when the therapeutic privilege was more widely accepted, physicians were often wrong in their assumptions about the harm that disclosure would cause. For example, in a 1961 study, 90% of physicians preferred *not* to tell a cancer patient their diagnosis because they anticipated the profoundly disturbing psychological effects of providing a terminal diagnosis.^72^ However, further investigation of this patient cohort failed to uncover any evidence supporting the clinicians' attitudes, including poor psychological outcomes, like suicide, in those who were disclosed terminal diagnoses.^73^ The lesson here is that clinicians who invoke the therapeutic privilege should have a clear and concrete understanding of exactly what harm is avoided through nondisclosure. The less certain and less imminent the perceived harm is, the less support the possibility of such harms provide in favour of the therapeutic privilege.

Third, it may be possible to mitigate at least some of the harms associated with disclosure, which we shall now outline in more detail.

#### 2.4.1 Stigma

A significant concern for ARMS patients is the risk of stigma and discrimination. These concerns are grounded in the belief that ARMS patients will be stigmatised due to the ARMS label's relationship with psychotic disorders. Notably, whilst stigma can be shown by third parties, people with mental illness can also internalise stigma in instances of ‘self‐stigma’; Colizzi et al. outline evidence suggesting that ARMS patients may be at risk of both kinds of stigma.^74^ Of course, concerns about stigma in this context are also amplified by the notion that nontransitioners will *unnecessarily* suffer long‐term consequences of stigma and discrimination.^75^ Given the severity of the harms associated with stigma, it is important that steps are taken to mitigate this risk. One way of mitigating the risk of public stigma is to ensure that ARMS diagnoses occur in healthcare settings where clinician–patient confidentiality applies. However, this is not a panacea—it does not address the issue of self‐stigma, and it is not unusual for individuals and caregivers to communicate a diagnosis with others.

However, it is important to distinguish between stigma that is *associated* with ARMS patients and stigma that is *caused as a result* of disclosing an ARMS label to patients.^76^ Empirical studies suggest a clear difference between the two: Yang et al. found that patients described stigma as being more associated with their symptoms than the label itself,^77^ while Woodberry et al. found that ARMS patients reported significantly *less* negative emotion after being disclosed their psychosis risk status.^78^ In a similar vein, Colizzi et al. suggest that diagnostic labelling may even confer considerable benefit as the clinician has the opportunity to clarify the patient's concerns.^79^

These studies suggest that ARMS disclosure does not uniformly cause harm in all patients. The varied outcomes of different studies instead likely reflect the various emotional reactions that one would expect after being diagnosed with a serious mental health condition. However, withholding disclosure to all patients on the basis of a concern that some patients might potentially feel stigmatised would be unjustified, considering the various reasons in favour of disclosure discussed above.

Nonetheless, stigma patently exists, and it is a harm that should be actively screened and minimised in all patients. It is also a harm that lends support to the thought that patients should be able to exercise an RNTK. Encouragingly, strategies to minimise stigma are being actively explored, including cognitive therapies^80^ and service‐level changes such as setting up general youth mental health clinics rather than hospital‐based care.^81^ The provision of accurate information has also been linked to less stigmatising attitudes, supporting the notion that psychoeducation can contribute to the minimisation of harm.^82^

#### 2.4.2 Disclosure leading to harmful interventions

The potential for ARMS patients to be prescribed potentially harmful interventions like antipsychotics is another concern associated with ARMS disclosure.^83^

However, whilst an ARMS diagnosis might typically be necessary for a patient to access treatment, disclosure of such a diagnosis need not automatically imply that a patient will (or indeed ought to) receive treatment.^84^

Indeed, antipsychotics are *not* routinely recommended as the first‐line treatment for ARMS patients,^85^ and are only recommended thereafter under specific circumstances such as severe and progressive symptoms or if there is a risk of self‐harm or aggression.^86^ Therefore, disclosure resulting in the use of interventions that cause more harm than benefit should not occur if clinicians adhere appropriately to clinical treatment guidelines.

Yet, empirical data do lend credence to concerns of inappropriate antipsychotic use. Researchers in both North America and Australia have found that 23%–27% of ARMS patients are already prescribed antipsychotics by the time they are referred to psychosis risk services.^87^ In effect, this potential harm is one that has a theoretically clear solution (to follow clinical guidelines), but this may be difficult to implement in practice.

## 3 A FRAMEWORK FOR DISCLOSURE

So far, our ethical analysis has evaluated the relative normative significance of the ethical values that favour and oppose ARMS disclosure. We summarise each argument in Table 1 and now use these arguments to build an ethical framework that informs a coherent disclosure strategy for different ARMS patients.

**Table 1:** Ethical principles and factors that favour and oppose disclosure, including how each factor may have decreased normative weight

Ethical principles *against* disclosure	Ethical principles *favouring* disclosure
Nonmaleficence Autonomy (preference not to know)	Autonomy (enhanced understanding) Autonomy (preference to know) Beneficence
Factors that *oppose* disclosure	Factors that *favour* disclosure
Labelling of patients may increase feelings of stigma and discrimination If labelled at risk of psychosis, patients may be more vulnerable to inappropriate use of harmful interventions like antipsychotics Patients may have a preference not to know	Disclosure enhances understanding so that patients can make autonomous decisions in line with their values Patients may have a preference to know information Disclosure provides a source of explanation and psychological relief to the patient Disclosure facilitates opportunistic education and may strengthen engagement with mental health services
How can these factors have decreased normative weight?	How can these factors have decreased normative weight?
Stigma‐reducing strategies and policies at the level of clinical interaction and service provision Appropriate risk assessment of potential harms Judicious use of interventions following clinical guidelines and prescribed by specialist clinician	Patient has compromised capacity (age, comorbid condition, etc.) Nonexpert provides diagnosis Manner/timing of diagnosis and explanation impair understanding Lack of high‐quality evidence for interventions

### 3.1 Taking stock and synthesising an overall disclosure strategy

Our above analysis demonstrates that there are often very strong moral reasons in favour of full disclosure of an ARMS label to patients who have fulfilled diagnostic criteria via the CAARMS. These reasons are primarily grounded in considerations of autonomy and beneficence. The information that an ARMS disclosure conveys is material to a wide range of decisions that the individual faces, both inside and outside of the immediate medical encounter. Furthermore, as detailed above, empirical evidence suggests that most ARMS patients who attend specialist clinics would like to know more information about their psychosis risk. Finally, we have also outlined various benefits that are associated with ARMS disclosure, including disclosure acting as a source of explanation for a nebulous collection of symptoms that is causing significant clinical distress.

Despite this strong theoretical position, these arguments in favour of disclosure are defeasible. Many of the arguments in favour of disclosure are premised on the assumption that the individual will understand the information that is being disclosed. Perhaps more significantly, there are some situations in which disclosure could lead to foreseeable harms that in extreme cases could plausibly outweigh the reasons favouring disclosure, or where patients wish to exercise an RNTK.

The strength of these competing moral reasons will typically depend on patient‐specific factors that require careful assessment of individual cases. With that in mind, we have created a principle‐based flowchart that clinicians might refer to that guides case‐by‐case decision‐making about disclosure in this context. These questions may guide the clinician to consider which ethical principles best justify a particular disclosure strategy, where full disclosure amounts to fully disclosing an ARMS label to patients. The intent of the flowchart is not to replace measured ethical reflection, which will always require careful consideration of each individual's circumstances, but to function as an ethical aid when the clinician is uncertain about how to decide whether disclosure is ethically justified.

Our analysis shares some similarities with Mittal et al.'s discussion of disclosure strategies in this context. They analysed three models of disclosure (full, partial and nondisclosure) eventually advocating for a hybrid model of disclosure according to which adult patients and parents/legal guardians will typically be given full disclosure and underage patients will be given full or partial disclosure depending on case‐specific factors.^88^ However, they note that there are some caveats to this general framework in some nontypical cases.

On this model, full disclosure is understood to include informing the patient about their ARMS status as well as explaining important features about the label, including the transition rate to psychosis, other clinical trajectories and any associated symptoms. In contrast, under their partial disclosure model,

the clinician … would not explicitly provide the APS [ARMS] diagnosis, but rather suggest that the suspiciousness and perceptual abnormalities concern experiences that may reflect thought disorder (a collection of symptoms that sometimes progresses into more serious mental illness).^89^

We are sympathetic to Mittal et al.'s hope that the partial disclosure might serve to balance different moral principles that are operative in this context. Nonetheless, our framework diverges from Mittal et al.'s model in two key ways.

First, we have some reservations about how partial disclosure might be worded on their approach. In order to maintain some respect for patients' autonomy, Mittal et al. suggest that partial disclosure might involve informing patients that their symptoms reflect a ‘thought disorder’, described as a ‘collection of symptoms that sometimes progresses into more serious mental illness’.^90^ Of course, one problem with this somewhat incomplete explanation is that it runs the risk of mitigating a patient's ability to make a more informed decision about their future, especially if the patient and/or guardian have misconceptions about psychosis. Naturally, the rationale for running this risk with a partial disclosure strategy is that such limited disclosure may be less likely to harm the patient insofar as it involves withholding a potentially stigmatising diagnostic label. However, we are not convinced that Mittal et al.'s suggested wording of the partial disclosure model would achieve this aim. After all, ‘thought disorder’ is conventionally accepted as a defining feature of psychosis,^91^ and thus may itself raise concerns about stigmatisation. Furthermore, given that it is accepted as a defining feature of psychosis, such disclosure may cause further confusion or distrust in patients who later seek more information from alternative sources about their diagnosis. We are thus concerned that rather than forging a balance between the principles of nonmaleficence and autonomy, this form of partial disclosure may in some ways achieve the worst of both worlds.

That said, we are open to there being some form of partial disclosure in this context that could successfully mitigate stigma while sufficiently informing the patient. For instance, partial disclosure might simply relate to the current symptoms that the patient is experiencing, and whether or not those symptoms are likely to ameliorate.

We diverge more significantly from Mittal et al.'s approach in denying that age alone, or indeed the possession of institutional capacity (to make decisions pertaining to the management of symptoms), should serve as a reliable proxy for what will constitute the ethically justified disclosure strategy in most cases. As Mittal et al. themselves note, there may be cases in which an underage patient should be given full disclosure, and we agree that this should be assessed on a case‐by‐case basis. However, we would further contend that there could feasibly be cases in which there might be sufficient ethical reasons to refrain from providing full disclosure to an adult patient, and that this also should be assessed on a case‐by‐case basis.

This is not to deny that the possession of institutional capacity has no bearing on the moral analysis. Typically, the treatment preferences of individuals with the institutional capacity to make decisions about the management of a medical condition are taken to have greater significance than the preferences of those who lack such capacity. For instance, individuals with institutional capacity may be afforded the right to refuse treatment that has been determined to be in their best interests; in contrast, the wishes of patients lacking institutional capacity might not similarly be respected. In a similar vein, in the current context, it might be argued that the preference to know (or not know) information has greater moral significance if the patient has been deemed to have institutional capacity to make determinative decisions about their treatment. In contrast, whilst the preferences of a patient lacking institutional capacity to make such decisions are plausibly relevant to an overall assessment of whether disclosure will be in their best interests, it might be argued that these preferences might yet be paternalistically outweighed by other prudential considerations. This is comparable to the manner in which the treatment preferences of those lacking institutional capacity can sometimes be overridden in order to protect their best interests elsewhere in medicine.

For this reason, we agree with a feature that is implicit in Mittal et al.'s model; an individual's possession of institutional capacity to make treatment decisions about the management of their condition has important implications for appropriate disclosure strategies. Crucially though, in some cases, the same moral questions will be relevant for finally determining the ethically justified disclosure strategy regardless of whether the patient has or lacks institutional capacity. This is why the two sides of our flowchart eventually recombine on the question of whether full disclosure is necessary for achieving certain benefits.

On our approach, partial disclosure will only be justified in a limited number of cases, where case‐by‐case analysis has shown that there is significant uncertainty about the balance between considerations of autonomy, beneficence and nonmaleficence. Crucially though, on our model, this same degree of uncertainty can be generated both in cases where a patient has institutional capacity and cases where such capacity is absent.

However, we should not overstate the difference between our model and Mittal et al.'s. Indeed, these authors' hybrid approach does call for a case‐by‐case analysis for some patients, and they highlight important caveats in their concluding section. Our approach can be understood to provide a framework for guiding the case‐by‐case ethical analysis that these authors also highlight as necessary in this context and that directly engages with the underlying values at stake in these decisions. Another advantage of this principle‐based approach is that it can accommodate the prospect that the strength of the different reasons favouring or opposing disclosure may change. For example, clinicians from different cultures can ascribe different degrees of normative significance to each principle. Additionally, future scientific developments such as the improved identification of biomarkers in ARMS patients can be integrated and weighed up within our framework.^92^

### 3.2 Practical recommendations to maximise ethical disclosure

Another use of our principle‐based framework is to consider how each bioethical principle can provide guidance on how to maximise ethical disclosure in a clinical setting. Here, we consider each analysed principle in turn to provide practical recommendations for ARMS clinicians.

To ensure maximal understanding, the clinician should first ascertain whether specific patient circumstances may impact understanding. Legal minors should be consulted in tandem with their guardians to explore all parties' concerns and clarify any misconceptions after carefully disclosing their risk status and the implications of an ARMS diagnosis. Disclosure should also be provided in such a way as to minimise the complexity and quantity of information, with opportunities for patients to clarify their understanding and ask questions. Additionally, clinicians should have sufficient expertise to ensure that they can accurately diagnose and disclose relevant information about the ARMS label.

Respecting patients' preferences is a more difficult principle to adhere to in clinical practice. This is because any preference not to know one's ARMS diagnosis cannot be wholly ascertained before disclosure of *some* psychosis risk has been made to the patient. To respect a potential preference not to know, we suggest that the clinician should explicitly gain the patient's consent to carry out the CAARMS, which assesses for not only psychosis risk but also other mental disorders. Consequently, the patient would be aware that they are at some risk of psychosis and that tests are available that can more accurately stratify their psychosis risk, in line with our RNTK analysis in Section 2.2.

Adhering to beneficence can be achieved through a measured, clear and hopeful disclosure of information, emphasising that although the patient is at a higher risk than the general population for psychosis risk, most patients do not transition to psychosis. Clinicians could also discuss with patients that those who engage earlier with mental health services with their risk status have more favourable prognoses.^93^

Finally, harm minimisation can occur at both a clinical and a systemic level. Clinically, stigma minimisation may be carried out by ascertaining the patient's (and guardian's) preconceived understanding of psychosis and mental health. On a systemic level, more research into stigma and psychosis risk can highlight further areas for improvement in stigma minimisation, such as being aware that some cultural populations may be more likely to harbour stigmatising attitudes towards individuals at risk of psychosis.^94^ One other avenue to minimise stigma is to directly ask patients what diagnostic label they find least stigmatising.^95^

### 3.3 Limitations of our framework

**Figure 1:**
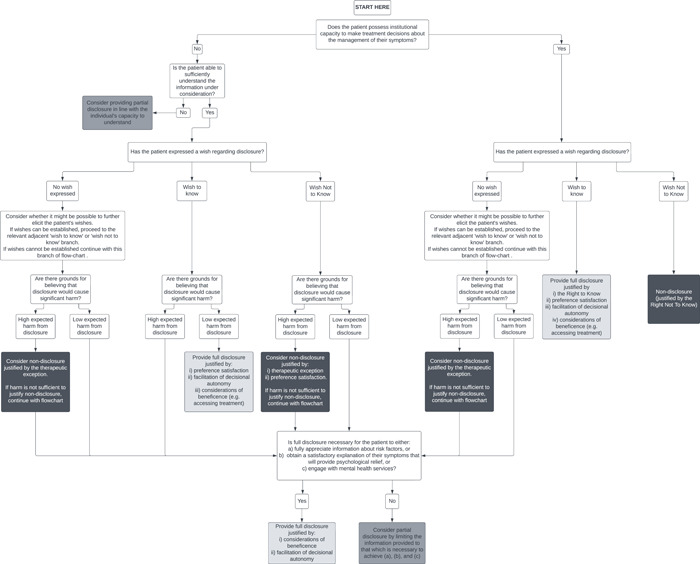
A flowchart to guide clinicians when considering disclosure of a psychosis risk label or diagnosis. The three outcomes include full disclosure (light grey), partial disclosure (dark grey) or nondisclosure (black).

One limitation of our framework is that we did not specifically focus on distributive justice within our principle‐based analysis. This is because the original impetus for our paper stemmed from debate about whether ARMS disclosure was ethically justifiable to diagnosed patients in specialised settings, where justice considerations are less applicable. However, some have argued that health resources are better allocated to other preventive health approaches such as the prevention of cannabis use.^96^ If preventive psychiatry moves towards a more ‘transdiagnostic’ approach to at‐risk states,^97^ the fundamental principles influencing disclosure would not change, but the normative significance of each principle would change, requiring fresh ethical analysis (Figure 1).

## 4 CONCLUSION

Due to our current scientific understanding and the unique characteristics of ARMS patients, disclosing an ARMS label is fraught with ethical pitfalls and dilemmas. To address this, we developed a disclosure strategy grounded in a principle‐based ethical analysis that we have operationalised in a flowchart to aid clinicians in ethical decision‐making regarding ARMS disclosure. We believe that there are strong autonomy‐ and beneficence‐based reasons in favour of disclosure. However, we also acknowledge that some situations may weaken reasons in favour of disclosure or strengthen reasons against disclosure, requiring adaptions in a clinician's ARMS disclosure strategy. Our framework for ARMS disclosure grounded in these principles should assist clinicians in deciding how to approach ethical disclosure to ARMS individuals.

## CONFLICT OF INTEREST

The authors declare no conflict of interest.

## Footnotes

1 Zachar, P., First, M. B., & Kendler, K. S. (2020). The DSM‐5 proposal for attenuated psychosis syndrome: A history. *Psychological Medicine, 50*(6), 920–926. Note that this prospective category is different to the strictly retrospective category of the schizophrenia prodrome. People are only considered to have exhibited the schizophrenia prodrome if they have gone on to be diagnosed with schizophrenia.

2 An example of attenuated hallucinations versus typical hallucinations are sounds (e.g., rumbling) that are interpreted as puzzling or unusual, and where ‘skepticism about their reality can still be induced’. See pp. 783–784 of American Psychiatric Association. (2013). *Diagnostic and statistical manual of mental disorders* (5th ed.). American Psychiatric Association.

3 Fusar‐Poli, P., Borgwardt, S., Bechdolf, A., Addington, J., Riecher‐Rossler, A., Schultze‐Lutter, F., Keshavan, M., Wood, S., Ruhrmann, S., Seidman, L. J., Valmaggia, L., Cannon, T., Velthorst, E., De Haan, L., Cornblatt, B., Bonoldi, I., Birchwood, M., McGlashan, T., Carpenter, W., … Yung, A. (2013). The psychosis high‐risk state: A comprehensive state‐of‐the‐art review. *JAMA Psychiatry, 70(1)*, 107–120. https://doi.org/10.1001/jamapsychiatry.2013.269

4 Another diagnostic test, the Structured Interview for Psychosis‐Risk Syndrome, has similar diagnostic validity to the CAARMS. We will refer to the CAARMS in this paper as the default diagnostic test for an ARMS diagnosis for simplicity in a bioethical context. See Fusar‐Poli, P., Cappucciati, M., Rutigliano, G., Lee, T. Y., Beverly, Q., Bonoldi, I., Lelli, J., Kaar, S. J., Gago, E., Rocchetti, M., Patel, R., Bhavsar, V., Tognin, S., Badger, S., Calem, M., Lim, K., Kwon, J. S., Perez, J., & McGuire, P. (2016). Towards a standard psychometric diagnostic interview for subjects at ultra high risk of psychosis: CAARMS versus SIPS. *Psychiatry Journal*, 2016, 7146341. https://doi.org/10.1155/2016/7146341

5 While we have chosen to refer to the psychosis high risk state with the term ‘ARMS’, rather than the DSM‐5 term ‘Attenuated Psychosis Syndrome’ (APS), or other terms like ‘Ultra High Risk’ and ‘Clinical High Risk’, we believe that these labels operate similarly enough for the purposes of our bioethical analysis. There are some technical differences between a DSM‐5‐APS diagnosis and an ARMS diagnosis; Most notably, the method of assessment, including whether specific psychometric training is needed to carry out the assessment; See de Pablo, G. S., Catalan, A., & Fusar‐Poli, P. (2020). Clinical validity of DSM‐5 attenuated psychosis syndrome: Advances in diagnosis, prognosis, and treatment. *JAMA Psychiatry*, *77*(3), 311–320.  https://doi.org/10.1001/jamapsychiatry.2019.3561 for further reading. Inconsistency in terminology when referring to the psychosis high risk state is a recurring point of contention in the literature; See section 7 in Woods, S. W., Bearden, C. E., Sabb, F. W., Stone, W. S., Torous, J., Cornblatt, B. A., Perkins, D. O., Cadenhead, K. S., Addington, J., Powers 3rd, A. R., Mathalon, D. H., Calkins, M. E., Wolf, D. H., Corcoran, C. M., Horton, L. E., Mittal, V. A., Schiffman, J., Ellman, L. M., Strauss, G. P., … Anticevic, A. (2020). Counterpoint. Early intervention for psychosis risk syndromes: Minimizing risk and maximizing benefit. *Schizophrenia Research, 227, 10–17*. https://doi.org/10.1016/j.schres.2020.04.020 for further information.

6 Yung, A. R., Pan Yuen, H., Mcgorry, P. D., Phillips, L. J., Kelly, D., Dell'Olio, M., Francey, S. M., Cosgrave, E. M., Killackey, E., Stanford, C., Godfrey, K., & Buckby, J. (2005). Mapping the onset of psychosis: The comprehensive assessment of at‐risk mental states. *Australian & New Zealand Journal of Psychiatry*, *39*(11–12), 964–971.

7 Corcoran, C., Malaspina, D., & Hercher, L. (2005). Prodromal interventions for schizophrenia vulnerability: the risks of being “at risk”. *Schizophrenia Research*, *73*(2–3), 173–184; Corcoran, C. M. (2016). Ethical and epidemiological dimensions of labeling psychosis risk. *AMA Journal of Ethics*, *18*(6), 633.

8 Malhi, G. S., Bell, E., Hamilton, A., & Morris, G. (2021). Early intervention for risk syndromes: What are the real risks? *Schizophrenia Research*, *227*, 4–9.

9 Fusar‐Poli, P., Cappucciati, M., Rutigliano, G., Schultze‐Lutter, F., Bonoldi, I., Borgwardt, S., Riecher‐Rössler, A., Addington, J., Perkins, D., Woods, S. W., McGlashan, T. H., Lee, J., Klosterkötter, J., Yung, A. R., & McGuire, P. (2015). At risk or not at risk? A meta‐analysis of the prognostic accuracy of psychometric interviews for psychosis prediction. *World Psychiatry*, *14*(3), 322–332. https://doi.org/10.1002/wps.20250. It should be noted that the reasons underlying the low (and, indeed, decreasing) transition rate are poorly understood. As a reviewer points out, it may be that low conversion rates to psychosis are not indicative of ‘false positives’ per se, but rather that receiving diagnostic information has made some difference in mitigating conversion. If this explanation is correct, then this would add to the beneficence‐based argument in favour of disclosure outlined below. However, there are other competing explanations, including the possibility that the pretest population in empirical studies that assess for the transition rate in ARMS individuals have been ‘diluted’ (See: Yung, A. R., Nelson, B., Stanford, C., Simmons, M. B., Cosgrave, E. M., Killackey, E., Phillips, L. J., Bechdolf, A., Buckby J., & McGorry, P. D. (2008). Validation of “prodromal” criteria to detect individuals at ultra high risk of psychosis: 2 year follow‐up. *Schizophrenia Research*, *105*(1), 10–17. https://doi.org/10.1016/j.schres.2008.07.012). We lack the space to argue in favour of the best explanation of this phenomenon, so we shall remain agnostic for the purposes of our argument.

10 Polari, A., Lavoie, S., Yuen, H.‐P., Amminger, P., Berger, G., Chen, E., deHaan, L., Hartmann, J., Markulev, C., Melville, F., Nieman, D., Nordentoft, M., Riecher‐Rössler, A., Smesny, S., Stratford, J., Verma, S., Yung, A., McGorry, P., & Nelson, B. (2018). Clinical trajectories in the ultra‐high risk for psychosis population. *Schizophrenia Research*, *197*, 550–556. https://doi.org/10.1016/j.schres.2018.01.022

11 Colizzi, M., Ruggeri, M., & Lasalvia, A. (2020). Should we be concerned about stigma and discrimination in people at risk for psychosis? A systematic review. *Psychological Medicine*, *50*(5), 705–726. https://doi.org/10.1017/S0033291720000148; Jorm, A. F. (2012). Ethics of giving antipsychotic medication to at‐risk young people. *Australian & New Zealand Journal of Psychiatry*, *46*(9), 908–909.

12 Kuharic, D. B., Kekin, I., Hew, J., Kuzman, M. R., & Puljak, L. (2019). Interventions for prodromal stage of psychosis. *Cochrane Database of Systematic Reviews*, (11).

13 Cassetta, B. D., & Goghari, V. M. (2014). Ethical considerations of screening and early intervention for clinical high‐risk psychosis. *Ethics & Behavior*, *25*(1), 1–20. https://doi.org/10.1080/10508422.2014.880920

14 Early Psychosis Guidelines Writing Group, & EPPIC National Support Program. (2016). *Australian clinical guidelines for early psychosis* (2nd ed., p. 7). The National Centre of Excellence in Youth Mental health. https://www.orygen.org.au/Campus/Expert-Network/Resources/Free/Clinical-Practice/Australian-Clinical-Guidelines-for-Early-Psychosis/Australian-Clinical-Guidelines-for-Early-Psychosis.aspx

15 Addington, J., Addington, D., Abidi, S., Raedler, T., & Remington, G. (2017). Canadian treatment guidelines for individuals at clinical high risk of psychosis. *The Canadian Journal of Psychiatry*, *62*(9), 656–661.

16 See p. 413 of Schultze‐Lutter, F., Michel, C., Schmidt, S. J., Schimmelmann, B. G., Maric, N. P., Salokangas, R. K., Riecher‐Rössler, A., van der Gaag, M., Nordentoft, M., Raballo, A., Meneghelli, A., Marshall, M., Morrison, A., Ruhrmann, S., & Klosterkotter, J. (2015). EPA guidance on the early detection of clinical high risk states of psychoses*. European Psychiatry*, *30*(*3)*, 405–416. https://doi.org/10.1016/j.eurpsy.2015.01.010

17 Benoit, L., Russo, T., Barry, C., Falissard, B., & Henckes, N. (2019). “You have to believe in something”: Risk of psychosis and psychiatrists' beliefs in the self‐fulfilling prophecy. *Social Science & Medicine*, *230*, 20–29.

18 Corcoran, op. cit. note 7, p. 634; Woods, S. W., et al., op. cit. note 5, p. 14; Mittal, V. A., Dean, D. J., Mittal, J., & Saks, E. R. (2015). Ethical, legal, and clinical considerations when disclosing a high‐risk syndrome for psychosis. *Bioethics*, *29*(8), 543–556. https://doi.org/10.1111/bioe.12155

19 Corcoran, op. cit. note 7, p. 635.

20 See pp. 131–133, Beauchamp, T., & Childress, James F. (2013). *Principles of biomedical ethics* (7th ed.). Oxford University Press.

21 See p. 161 of Pugh, J. (2020). *Autonomy, rationality, and contemporary bioethics*. Oxford University Press.

22 Mittal, V. A, et al., op. cit. note 18, p. 545.

23 Ibid: 549.

24 Ibid: 553.

25 Montgomery v. Lanarkshire Health Board, 2015 U.K.S.C. 11 (2015).

26 Liddell, K., Skopek, J. M., Le Gallez, I., & Fritz, Z. (2022). Differentiating negligent standards of care in diagnosis. *Medical Law Review*, *30*(1), 33–59.

27 Corcoran, C., et al. (2005), op. cit. note 7, p. 6.

28 Fusar‐Poli, P., et al., op. cit. note 9, p. 326.

29 Rutigliano, G., Valmaggia, L., Landi, P., Frascarelli, M., Cappucciati, M., Sear, V., Rocchetti, M., De Micheli, A., Jones, C., Palombini, E., McGuire, P., & Fusar‐Poli, P. (2016). Persistence or recurrence of non‐psychotic comorbid mental disorders associated with 6‐year poor functional outcomes in patients at ultra high risk for psychosis. *Journal of Affective Disorders*, *203*, 101–110.

30 Our use of the term ‘ARMS negative’ refers to suspected ARMS patients who have been assessed by a specialist ARMS clinician but who do not meet ARMS criteria after CAARMS assessment.

31 Fusar‐Poli, P., op. cit. note 9, p. 326.

32 Fusar‐Poli, P., Rutigliano, G., Stahl, D., Davies, C., De Micheli, A., Ramella‐Cravaro, V., Bonoldi, I., & McGuire, P. (2017). Long‐term validity of the At Risk Mental State (ARMS) for predicting psychotic and non‐psychotic mental disorders. *European Psychiatry*, *42*, 49–54.

33 Fusar‐Poli, P., Cappucciati, M., Borgwardt, S., Woods, S. W., Addington, J., Nelson, B., Nieman, D. H., Stahl, D. R., Rutigliano, G., Riecher‐Rössler, A., Simon, A. E., Mizuno, M., Lee, T. Y., Kwon, J. S., Lam, M. M., Perez, J., Keri, S., Amminger, P., Metzler, S, … McGuire, P. K. (2016). Heterogeneity of psychosis risk within individuals at clinical high risk: A meta‐analytical stratification. *JAMA Psychiatry*, *73*(2), 113–120. https://doi.or/10.1001/jamapsychiatry.2015.2324

34 Corcoran, C. M., Mittal, V. A., & Woods, S. W. (2021). Attenuated psychosis syndrome should be moved to the main section in DSM‐5‐TR. *JAMA Psychiatry*, *78*(8), 821–822.

35 Fulford, K., & Radoilska, L. (2012). Three challenges from delusion for theories of autonomy. In Radoilska, L. (Ed.), *Autonomy and mental disorder* (pp. 44–74). Oxford University Press.

36 Markova, I., & Berrios, G. E. (1992). The meaning of insight in clinical psychiatry. *The British Journal of Psychiatry*, *160*(6), 850–860.

37 Ruissen, A., Widdershoven, G., Meynen, G., Abma, T., & Van Balkom, A. (2012). A systematic review of the literature about competence and poor insight. *Acta Psychiatrica Scandinavica*, *125*(2), 103–113.

38 In this case, we are assuming patients have capacity at baseline. Patients who lack capacity at baseline, whether due to age or comorbid disorder, will be addressed in our next section.

39 Wilhelms, E. A., & Reyna, V. F. (2013). Effective ways to communicate risk and benefit. *The Virtual Mentor: VM*, *15*(1), 34.

40 See pp. 276–277 of Faden, R. R., & Beauchamp, T. L. (1986). *A history and theory of informed consent*. Oxford University Press.

41 For individuals below the age of 16 in the United Kingdom, there is a further question of whether ARMS patients can attain Gillick competence (where children under 16 in England and Wales can consent to medical treatment under certain conditions without parental permission), which is beyond the scope of this paper.

42 Diekema, D. S. (2003). Taking children seriously: What's so important about assent? *The American Journal of Bioethics*, *3*(4), 25–26. https://doi.org/10.1162/152651603322614481

43 Outram, S., Harris, G., Kelly, B., Cohen, M., Bylund, C. L., Landa, Y., Levin, T. T., Sandhu, H., Vamos, M., & Loughland, C. (2015). Contextual barriers to discussing a schizophrenia diagnosis with patients and families: Need for leadership and teamwork training in psychiatry. *Academic Psychiatry*, *39*(2), 174–180.

44 Corcoran, C. M., First, M. B., & Cornblatt, B. (2010). The psychosis risk syndrome and its proposed inclusion in the DSM‐V: A risk–benefit analysis. *Schizophrenia Research*, *120*(1), 16–22. https://doi.org/10.1016/j.schres.2010.03.018

45 Outram, S., et al., op. cit. note 43, pp. 178–180.

46 Corcoran, C. M., et al., op. cit. note 34, pp. 821–22.

47 Parascandola, M., Hawkins, J. S., & Danis, M. (2002). Patient autonomy and the challenge of clinical uncertainty. *Kennedy Institute of Ethics Journal*, *12*(3), 245–264.

48 Millum, J., & Bromwich, D. (2021). Informed consent: What must be disclosed and what must be understood? *The American Journal of Bioethics*, *21*(5), 46–58. https://doi.org/10.1080/15265161.2020.1863511

49 See p. 71 of Porter, K. M., Weiss, E. M., & Kraft, S. A. (2021). Promoting disclosure and understanding in informed consent: Optimizing the impact of the common rule “key information” requirement*. The American Journal of Bioethics*, *21*(5), 70–72.

50 Polari, A., et al., op. cit. note 10, p. 552.

51 Polari, A., Street, R., Conus, P., Finkelstein, A., Hartmann, J. A., Kim, S.‐W., McGorry, P., Schley, C., Simmons, M., Stratford, J., Thompson, A., Yung, A., Nelson, B., & Lavoie, S. (2021). Patients', carers' and clinicians' attitudes towards alternative terms to describe the at‐risk for psychosis state. *Schizophrenia Research*, *237*, 69–75.

52 Ibid: 73.

53 See Article 10(2) of Council of Europe. (1996). *Convention for the Protection of Human Rights and Dignity of Human Being with regard to the Application of Biology and Medicine: Convention on Human Rights and Biomedicine*. Directory of Legal Affairs, Strasbourg, 9.

54 Andorno, R. (2004). The right not to know: An autonomy based approach. *Journal of Medical Ethics*, *30*(5), 435–439; Bortolotti, L., & Widdows, H. (2011). The right not to know: The case of psychiatric disorders*. Journal of Medical Ethics*, *37*(11), 673–676; Davies, B., & Savulescu, J. (2021). The right not to know: Some steps towards a compromise. *Ethical Theory and Moral Practice*, *24*(1), 137–150. https://doi.org/10.1007/s10677-020-10133-9

55 Laurie, G. (2014). Recognizing the right not to know: Conceptual, professional, and legal implications. *Journal of Law, Medicine & Ethics*, *42*(1), 53–63.

56 Andorno, op. cit. note 54, p. 439.

57 Polari, A., et al., op. cit. note 51, p. 73.

58 Falkenberg, I., Valmaggia, L., Byrnes, M., Frascarelli, M., Jones, C., Rocchetti, M., Straube, B., Badger, S., McGuire, P., & Fusar‐Poli, P. (2015). Why are help‐seeking subjects at ultra‐high risk for psychosis help‐seeking? *Psychiatry Research*, *228*(3), 808–815. https://doi.org/10.1016/j.psychres.2015.05.018

59 An RNTK for psychosis risk based on Andorno's position may also be applicable to future developments in the investigation of biomarkers and psychosis risk.

60 Pate v. Threlkel, No. 84289, 661 278 (Fla: Supreme Court 1995).

61 ABC v. St George's Healthcare NHS Trust, 2020 455 (2020).

62 Mittal, V. A., et al., op. cit. note 18, p. 547.

63 Nelson, B., Amminger, G. P., Thompson, A., Wood, S. J., Yung, A. R., & McGorry, P. D. (2020). Commentary: Preventive treatments for psychosis: umbrella review (just the evidence). *Frontiers in Psychiatry*, *11*(488). p. 1. https://doi.org/10.3389/fpsyt.2020.00488

64 Kuharic, D. B., et al., op. cit. note 12, p. 2.

65 Blessing, A., Studer, A., Gross, A., Gruss, L. F., Schneider, R., & Dammann, G. (2017). Disclosure of diagnosis in early recognition of psychosis. *The Journal of Nervous and Mental Disease*, *205*(10), 757–761.

66 Hayne, Y. (2003). Experiencing psychiatric diagnosis: Client perspectives on being named mentally ill. *Journal of Psychiatric and Mental Health Nursing*, *10*(6), 722–729.

67 Machielsen, M., van der Sluis, S., & de Haan, L. (2010). Cannabis use in patients with a first psychotic episode and subjects at ultra high risk of psychosis: Impact on psychotic‐ and pre‐psychotic symptoms. *Australian & New Zealand Journal of Psychiatry*, *44*(8), 721–728. https://doi.org/10.3109/00048671003689710

68 Johnston, C., & Holt, G. (2006). The legal and ethical implications of therapeutic privilege‐is it ever justified to withhold treatment information from a competent patient? *Clinical Ethics*, *1*(3), 146–151.

69 Bortolotti, & Widdows, op. cit. note 54, p. 675.

70 Corcoran, C., et al., op. cit. note 7, p. 6.

71 Johnston & Holt, op. cit. note 68, p. 148.

72 Oken, D. (1961). What to tell cancer patients: a study of medical attitudes. *JAMA*, *175*(13), 1120–1128.

73 Ibid: 1126.

74 Colizzi, M., et al., op. cit. note 11, p. 705.

75 Raven, M., Stuart, G. W., & Jureidini, J. (2012). ‘Prodromal’ diagnosis of psychosis: Ethical problems in research and clinical practice. *Australian & New Zealand Journal of Psychiatry*, *46*(1), 64–65.

76 Corcoran, C. M., et al., op. cit. note 34, pp. 821–822.

77 Yang, L. H., Link, B. G., Ben‐David, S., Gill, K. E., Girgis, R. R., Brucato, G., Wonpat‐Borja, A. J., & Corcoran, C. M. (2015). Stigma related to labels and symptoms in individuals at clinical high‐risk for psychosis. *Schizophrenia Research*, *168*(1–2), 9–15.

78 Woodberry, K. A., Powers, K. S., Bryant, C., Downing, D., Verdi, M. B., Elacqua, K. M., … Yang, L. H. (2021). Emotional and stigma‐related experiences relative to being told one is at risk for psychosis. *Schizophrenia Research, 238*, 44–51. https://doi.org/10.1016/j.schres.2021.09.017

79 Colizzi, M., et al., op. cit. note 11, p. 723.

80 Baer, L. H., Shah, J. L., & Lepage, M. (2019). Anxiety in youth at clinical high risk for psychosis: A case study and conceptual model. *Schizophrenia Research*, *208*, 441–446. https://doi.org/10.1016/j.schres.2019.01.006

81 McGorry, P. D., Yung, A., & Phillips, L. (2001). Ethics and early intervention in psychosis: Keeping up the pace and staying in step. *Schizophrenia Research*, *51*(1), 17–29.

82 Yang, L. H., Anglin, D. M., Wonpat‐Borja, A. J., Opler, M. G., Greenspoon, M., & Corcoran, C. M. (2013). Public stigma associated with psychosis risk syndrome in a college population: Implications for peer intervention. *Psychiatric Services*, *64*(3), 284–288.

83 Jorm, op. cit. note 11, p. 908.

84 Corcoran, C. M., et al., op. cit. note 34, p. 821.

85 Early Psychosis Guidelines Writing Group & EPPIC National Support Program, op. cit. note 14, p. 31.

86 Ibid: 51.

87 Cannon, T. D. (2008). Neurodevelopment and the transition from schizophrenia prodrome to schizophrenia: Research imperatives. *Biological Psychiatry, 64(9)*, 737–738. https://doi.org/10.1016/j.biopsych.2008.07.027; Nelson, B. (2014). Attenuated psychosis syndrome: Don't jump the gun. *Psychopathology*, *47*(5), 292–296.

88 Mittal, V. A., et al., op. cit. note 18, pp. 554–556.

89 Ibid: 550.

90 Ibid: 550.

91 Hart, M., & Lewine, R. R. J. (2017). Rethinking thought disorder. *Schizophrenia Bulletin*, *43*(3), 514–522. https://doi.org/10.1093/schbul/sbx003

92 Corcoran, C. M., et al., op. cit. note 34, p. 821.

93 Correll, C. U., Galling, B., Pawar, A., Krivko, A., Bonetto, C., Ruggeri, M., Craig, T. J., Nordentoft, M., Srihari, V. H., Guloksuz, S., Hui, C. L. M., Chen, E. Y. H., Valencia, M., Juarez, F., Robinson, D. G., Schooler, N. R., Brunette, M. F., Mueser, K. T., Rosenheck, R. A., … Kane, J. M. (2018). Comparison of early intervention services vs treatment as usual for early‐phase psychosis: A systematic review, meta‐analysis, and meta‐regression. *JAMA Psychiatry*, *75*(6), 555–565. https://doi.org/10.1001/jamapsychiatry.2018.0623

94 He, E., Eldeeb, S. Y., Cardemil, E. V., & Yang, L. H. (2020). Psychosis risk stigma and help‐seeking: Attitudes of Chinese and Taiwanese residing in the United States. *Early Intervention in Psychiatry*, *14*(1), 97–105; Lee, E. H.‐M., Hui, C. L.‐M., Ching, E. Y.‐N., Lin, J., Chang, W.‐C., Chan, S. K.‐W., & Chen, E. Y.‐H. (2016). Public stigma in China associated with schizophrenia, depression, attenuated psychosis syndrome, and psychosis‐like experiences. *Psychiatric Services*, *67*(7), 766–770; Vyas, A., Wood, L., & McPherson, S. (2021). A qualitative exploration of stigma experiences of second‐generation British South‐Asian people using an early intervention in psychosis service. *Psychosis*, *13*(4), 302–314.

95 Polari, A., et al., op. cit. note 51, pp. 73–74.

96 Ajnakina, O., David, A. S., & Murray, R. M. (2019). ‘At risk mental state’ clinics for psychosis—An idea whose time has come–and gone! *Psychological Medicine*, *49*(4), 529–534; van Os, J., & Guloksuz, S. (2017). A critique of the “ultra‐high risk” and “transition” paradigm. *World Psychiatry*, *16*(2), 200–206. https://doi.org/10.1002/wps.20423

97 McGorry, P., & Nelson, B. (2016). Why we need a transdiagnostic staging approach to emerging psychopathology, early diagnosis, and treatment. *JAMA Psychiatry*, *73*(3), 191–192; McGorry, P. D., Hartmann, J. A., Spooner, R., & Nelson, B. (2018). Beyond the “at risk mental state” concept: Transitioning to transdiagnostic psychiatry. *World Psychiatry*, *17*(2), 133–142. https://doi.org/10.1002/wps.20514

